# Distribution Pattern of Ants in Huanglianshan National Nature Reserve From Yunnan, China

**DOI:** 10.1002/ece3.72404

**Published:** 2025-11-04

**Authors:** Xingze Li, Huiping Zeng, Yanhui Zhang, Yu Yu, Chao Chen

**Affiliations:** ^1^ Forestry College Southwest Forestry University Kunming Yunnan China; ^2^ Yunnan Huanglianshan National Nature Reserve Management and Protection Bureau Lvchun Yunnan China; ^3^ Kunming Natural History Museum of Zoology, Kunming Institute of Zoology Chinese Academy of Sciences Kunming Yunnan China

**Keywords:** ant diversity, distribution pattern, elevational gradients, habitat, Huanglianshan

## Abstract

To elucidate the distribution patterns of ant species in the Huanglianshan National Nature Reserve, a plot‐based survey method was employed to investigate ant diversity within the reserve. A total of 209 ant species, representing 9 subfamilies and 66 genera, were collected from 38 plots spanning eight vertical gradient transects. Elevation significantly influenced ant communities within the reserve. Both ant species richness and genus richness exhibited a unimodal pattern (peaking at mid‐elevations) rather than a simple linear relationship with increasing elevation. Ordination analysis revealed significant dissimilarity between communities at low‐ and high‐elevation sites. Furthermore, the distance‐decay model confirmed that community similarity significantly decreased with increasing elevational distance. Regression analysis demonstrated a significant positive correlation between the upper elevational distribution limit of ant species and their elevational distribution breadth, indicating that species occurring at higher elevations possess wider vertical adaptation ranges. Subsequent statistical analysis of foraging and nesting habits showed that the majority of ants foraged on the ground surface and nested within the soil. Among the habitats sampled, low‐altitude tropical seasonal rainforest and coniferous‐broadleaved mixed forest harbored the highest ant species richness. In contrast, eucalyptus plantations and rubber plantations, which exhibit a higher degree of landscape homogeneity, supported significantly fewer ant species. Our data establish a foundation for further research on elevational variation and provide context for discussing key aspects of ant management and conservation in Huanglianshan National Nature Reserve.

## Graphical abstract

This study found that ant diversity in the Huanglian Mountain National Nature Reserve exhibits a non‐linear distribution pattern along the altitudinal gradient, encompassing 209 species across 9 subfamilies and 66 genera. Elevation significantly structured ant communities in the reserve. Species and genus richness followed unimodal patterns (mid‐elevation peaks), while ordination revealed distinct low‐ vs. high‐elevation communities. Community similarity declined significantly with elevational distance. Species at higher elevations showed broader distribution ranges. Most ants foraged on the ground and nested in soil.

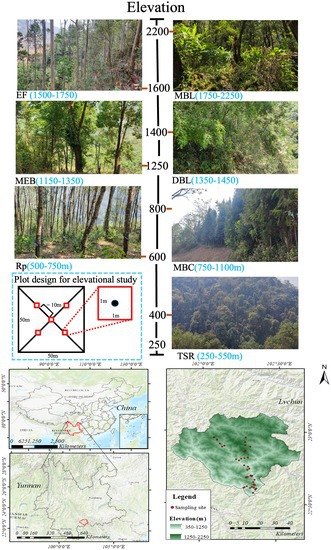

## 1 Introduction

Elevational gradients remain a primary focus in research exploring biodiversity responses to climatic conditions and their spatial variation patterns (Uhey et al. 2022; Schifani et al. 2024). They are generally recognized as major drivers of changes in species richness and distribution patterns. However, the influence of these gradients on species richness and distribution patterns varies substantially across different biological communities and geographical regions. This variability manifests in multiple dimensions, including community assembly structure, key underlying drivers, and the specific patterns of diversity–elevation relationships (Peters et al. 2016; Supriya et al. 2019). As critical global biodiversity hotspots and climate‐sensitive zones, tropical mountain ecosystems in transition zones hold significant value for revealing the ecological drivers of elevational gradients (Lasmar et al. 2020; Uhey et al. 2020). The Huanglianshan National Nature Reserve at the China‐Vietnam tropical border functions as a vital ecological corridor connecting the Yunnan Plateau and Southeast Asia. Its complex terrain with pronounced vertical variation sustains over 2000 unique species, including endemic species like the clouded leopard ( *Neofelis nebulosa* ) and the black crested gibbon ( *Nomascus concolor* ), while serving as a hotspot for discovering novel taxa and species distributions (Lin et al. 2024). Despite significant advances in vertebrate diversity research, particularly exemplified by studies on birds and fish (Mou et al. 2025; Liu et al. 2024; Song and Chen 2014), substantial knowledge gaps persist regarding the community assembly mechanisms and elevational ecological adaptation of invertebrates, particularly ants, in this region.

As the most diverse and abundant group of social insects worldwide, ants constitute a dominant component of insect fauna and hold considerable importance in ecosystem research (Carval et al. 2016; Kass et al. 2022). As typical small ectothermic organisms, ant populations exhibit graded responses to changes in environmental temperature through metabolic regulation, with their thermal tolerance thresholds directly constraining the boundaries of their geographical distributions (Shik et al. 2019; Couper et al. 2021). Moreover, most ant species are constrained by restricted dispersal capabilities and natural geographical barriers, rendering their migration processes highly susceptible to disruption by topographical features. Additionally, owing to their heightened sensitivity to habitat fragmentation and anthropogenic disturbances—often greater than that of other insect groups—the study of ant distribution patterns can accurately reflect the evolutionary trajectories of ecological processes under environmental stress (Jésica et al. 2025; Martello et al. 2018), thereby providing a unique perspective for elucidating biogeographical mechanisms.

Ant diversity along elevation gradients generally exhibits two typical distribution patterns: a decline in ant species richness with increasing elevation (He et al. 2020; Chen, Xion, et al. 2022) and a unimodal pattern, where species richness initially increases and then decreases with elevation (Kaspari et al. 2004). The observed variation in these distribution patterns is strongly associated with the climatic zones of mountain systems. In tropical mountains characterized by extensive species pools and intense competition, resource partitioning and narrow thermal tolerances frequently promote the formation of unimodal richness patterns (Longino et al. 2019; Nowrouzi et al. 2016). Conversely, in temperate mountains where environmental stressors prevail, severe physical filtering mechanisms (e.g., low temperature extremes) tend to generate monotonically decreasing species richness along elevational gradients (Bernadou et al. 2015). While this climatic zonation framework provides valuable macroecological insights, the principal drivers governing ant community distributions and their specific response mechanisms in particular mountain regions—especially protected areas with complex topography and heterogeneous vegetation spectra—remain inadequately investigated. Most existing studies have concentrated on mid‐latitude tropical regions (Ryder Wilkie 2010; Rocha‐Ortega et al. 2023), the northwestern Sichuan Plateau and the Yunnan Plateau in China (Guo, et al. 2022; Yin et al. 2023; Li, Xu, Li, et al. 2023; Li, Xu, Zhang, et al. 2023; Yang et al. 2024; He et al. 2022; Cui et al. 2023; You et al. 2025), as well as the Qinghai‐ Xizang Plateau (Chen, Xiong, et al. 2022; Yang et al. 2025). The latter studies indicate that ant species richness in China and Southeast Asia regions predominantly follows the first pattern, while the second pattern is more prevalent in high‐latitude and high‐altitude regions. The specific distribution characteristics are influenced by local habitat heterogeneity and the degree of human disturbance. However, the ecological adaptation mechanisms of ant communities along altitudinal gradients in the Huanglian Mountain National Nature Reserve still lack systematic research. This study, through a diversity survey of ant communities across different altitudes, aims to reveal the following ecological patterns: (1) to verify the response pattern of ant richness to altitudinal changes in low‐latitude subtropical regions; (2) to determine how the elevation adaptation range of ants varies at different altitudes in this region.

## 2 Materials and Methods

### 2.1 Overview of the Study Area

**FIGURE 1:**
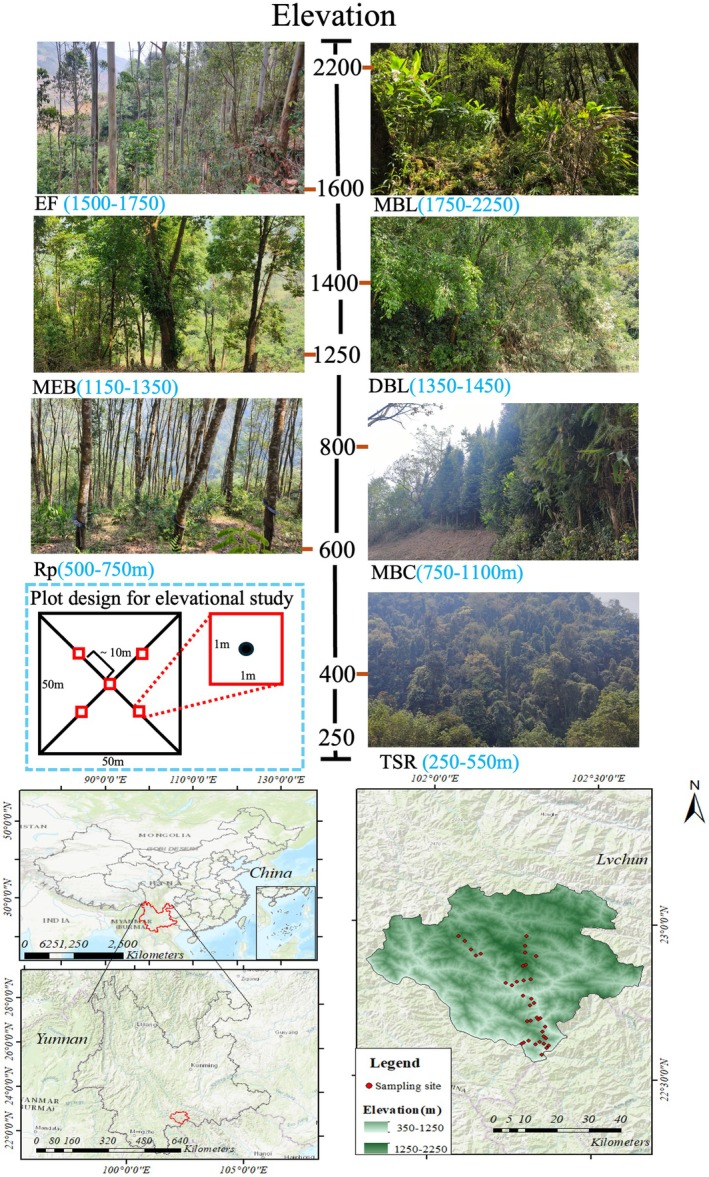
Overview of the study area. Abbreviations denote habitat types: DBL, deciduous broad‐leaved forest; ER, Eucalyptus forest; MBC, mixed broadleaf‐conifer forest; MBL, Mountain moss evergreen broad‐leaved forest; MEB, monsoon evergreen broadleaf forest; RP, rubber plantation; TSR, tropical seasonal rainforest.

The Huanglianshan National Nature Reserve in China is situated at the intersection of China, Vietnam, and Laos, forming part of the southern extension of the Ailao Mountains. Functioning as an “ecological stepping stone,” the reserve connects the Yunnan Plateau to the tropical forest of Southeast Asia (Figure 1) (Xu 2003). Its unique geographical position creates a transboundary ecological corridor. The temperature within the reserve decreases by 0.58°C for every 100 m increase in elevation, while precipitation displays a unimodal pattern with altitude. The highest precipitation occurs at mid‐high elevations (1000 m) with a peak value of 2350 mm, decreasing towards both lower and higher elevations (Myers et al. 2000). Eight transects were arranged from south to north within the study area, spanning an altitude range of 300 to 2250 m, covering the complete vertical zonation from tropical seasonal rainforest at the foot of the mountain to montane mossy evergreen broad‐leaved forest at the summit. This setup fully includes seven habitat types, such as rubber plantations and tropical seasonal rainforest, ensuring the systematicness and completeness of this survey in terms of both elevation gradient and habitat types (Figure 1).

### 2.2 Survey Sampling Methods

The plot survey method was used to investigate ants in the Huanglianshan National Nature Reserve from April 2023 to September 2024. This period covers both the dry and rainy seasons in Yunnan, allowing the study to reflect ant community responses to seasonal changes in moisture and ensuring that the data include their main activity periods. Within the reserve, at elevations ranging from 367 m to 2250 m, eight transects were laid out in a north–south direction. Along each transect, for every 250‐m increase in elevation, a 50‐m × 50‐m standard plot with typical regional vegetation and ecological characteristics was selected. In each plot, five 1‐m × 1‐m quadrats were set at equal intervals (10 m apart) along the diagonal, resulting in a total of 38 plots and 190 quadrats (Table 1). Within each quadrat, the species diversity of ants on the ground surface, in the soil layer, and in the canopy was investigated, and the number and frequency of ant species appearing in each transect were finally calculated. Due to field conditions, the actual elevation of plots may deviate by ±50 m. This vertical gradient design (250 m) effectively captures mountain environmental gradients (such as temperature, humidity, and vegetation), while the ±50‐m margin of error remains controlled, ensuring data comparability. After selecting quadrats, surface ants were surveyed first by collecting scattered individuals, which were placed into 2‐mL cryovials containing anhydrous ethanol after being labeled. If a nest was found, it was transferred to a plastic tray with a small spatula for counting, and up to 30 individuals representing all castes were collected and preserved separately, with the rest released. Next, soil layer ant surveys were conducted using a small pickaxe to excavate soil to a depth of 20 cm, collecting exposed individuals. If a nest was found in the soil, it was also counted, and specimens were collected. Finally, for the canopy survey, two people held a 2‐m × 2‐m white sheet above the quadrat, while another person vigorously shook the branches of shrubs and small trees above it to collect ants that fell onto the sheet. During the survey, both ant foraging sites and nesting sites were recorded. All collected ants were eventually brought back to the laboratory for specimen preparation and taxonomic identification.

**TABLE 1:** Characteristics of the eight vertical gradient transects.

Transects	Range of altitude/m	Number of habitats type
Dagu	350–1250	4
Erfu	500–1500	2
Xiaoheijiang	500–1250	2
Dongma	750–1500	1
Moluo	750–1250	2
Hexin	500–2250	4
Laobian	1750–1800	1
Qimaba	750–2000	4

### 2.3 Preparation and Identification of Specimens

**FIGURE 2:**
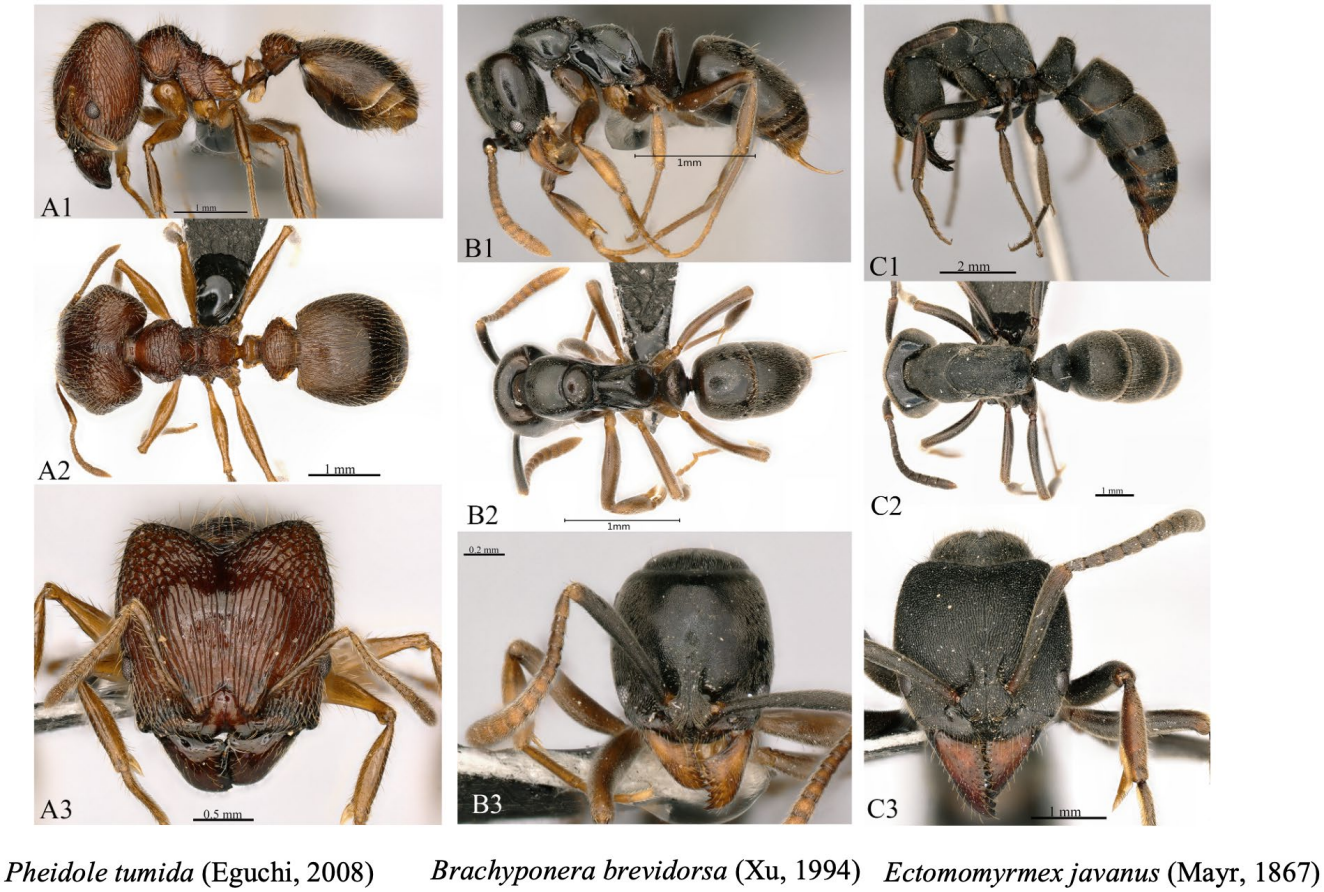
Three ant species characterized by wide distribution and high adaptability within the study area.

The ant specimens collected in the field are sorted, categorized, and numbered. For each specimen number (Figure 2), up to nine individuals are prepared as dry‐mounted specimens on triangular paper for identification purposes, while any additional individuals are preserved as wet specimens. The dry triangular paper specimens are classified and identified according to major domestic and international ant taxonomy monographs (Z. H. Xu 2002; Bolton 1994; Wu and Wang 1995; Zhou 2001).

### 2.4 Data Analysis

A Bray–Curtis dissimilarity matrix was constructed from species presence‐absence data and analyzed using principal coordinates analysis (PCoA) and non‐metric multidimensional scaling (NMDS) to decipher elevational variation patterns in ant community composition. We further constructed distance‐decay relationship (DDR) models via the vegan package to quantify shifts in community similarity across elevational gradients. Analysis of similarities (ANOSIM) tested the significance of group differences (Nowrouzi et al. 2016). All analyses were implemented in R (v3.1.0) using the iNEXT packages for Chao1 richness estimation, base stats for ANOVA and Tukey HSD tests, and the vegan packages for ordination analyses, DDR modeling, and ANOSIM testing (Clarke 1993; R Development Core Team 2009) (Supporting Information S4).

Using the lmer function from the lme4 R package (Bates et al. 2015), we fitted mixed‐effect models with polynomial terms to assess potential non‐linear relationships between elevation and two diversity measures: species richness and genus richness, while setting the sampling lins as a random factor.

The Jaccard coefficient value can quantify the similarity in species composition between biological communities, and the larger the coefficient value, the higher the sample similarity. Jaccard similarity coefficient: *J* [(*A*, *B*)] $=\frac{|A\cap B|}{|A\cup B|.}$

## 3 Results

### 3.1 Faunal Overview

**FIGURE 3:**
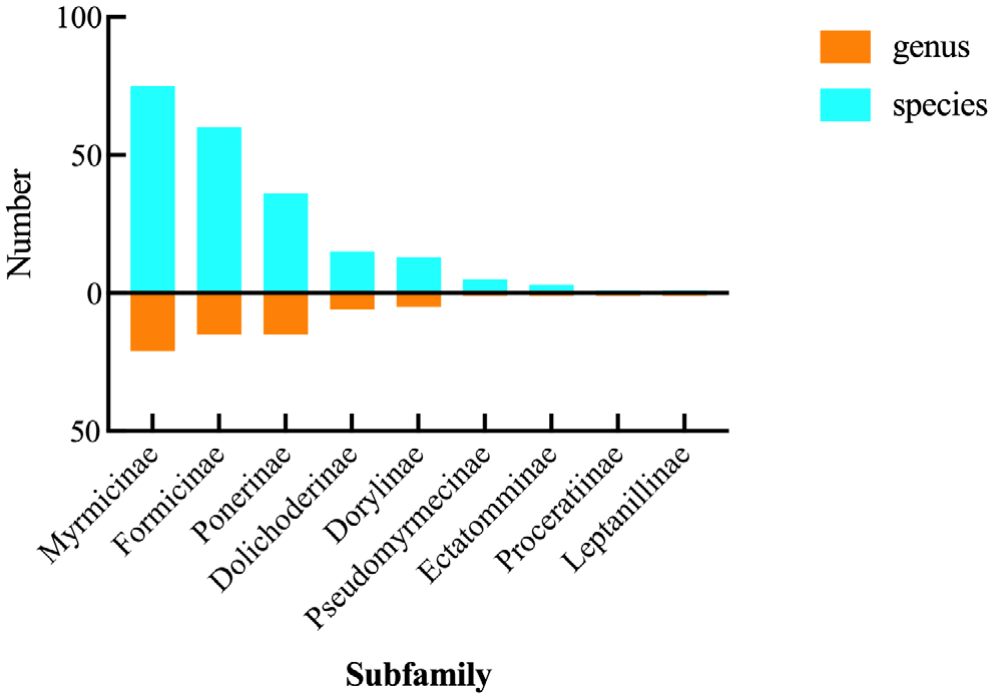
Abundance of ant families and genera.

A total of 30,604 ants were observed in eight transects and 38 plots within the Huanglianshan National Nature Reserve (Supporting Information S1). Upon identification, they were found to belong to 9 subfamilies, 66 genera, and 209 species (Figure 3), with an additional 24 undetermined species. Among them, the subfamily Myrmicinae included 21 genera and 75 species; Formicinae included 15 genera and 60 species; Ponerinae included 15 genera and 36 species; Dolichoderinae included 6 genera and 15 species; Dorylinae included 5 genera and 13 species; Pseudomyrmecinae included 1 genus and 5 species; Ectatomminae included 1 genus and 3 species; Proceratiinae included 1 genus and 1 species; and Leptanillinae included 1 genus and 1 species. The most widely distributed ant species were *Pheidole nodus* (Smith, 1874), *Crematogaster ferrarii* (Emery, 1887), *Camponotus mitis* (Smith, 1858), and *Nylanderia birmana* (Forel, 1902), all of which were found in all eight transects of Huanglianshan. Additionally, 21 species, including *Dolichoderus thoracicus* (Smith, 1860), *Ectomomyrmex javanus* (Mayr, 1867), and *Pheidole pieli* (Santschi, 1925) were found in seven transects. More than 63.22% of the ant species (132 species) were distributed in three or fewer transects, with 74 ant species (35.41%) found in only one transect (Supporting Information S2).

**FIGURE 4:**
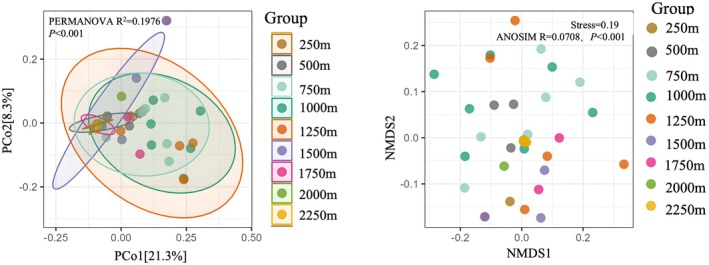
Ordination analyses of ant community composition across elevational gradients.

Both ordination analyses revealed significant elevational structuring in ant communities. Principal Coordinates Analysis (PCoA) (Figure 4, left) demonstrated distinct clustering along the first axis (PCo1: 21.3%), with low‐elevation sites (250–750 m) markedly separated from high‐elevation assemblages (> 1500 m). PERMANOVA confirmed that elevation explained 19.76% of compositional variation (*R* ^2^ = 0.1976, *p <* 0.001). Non‐metric Multidimensional Scaling (NMDS) (Figure 4 right; stress = 0.19) further indicated continuous species turnover along the elevational gradient. Although the grouping effect was weak, ANOSIM detected statistically significant differences among elevation zones (*R* = 0.0708, *p <* 0.001), suggesting gradual community shifts rather than discrete thresholds.

### 3.2 Elevational Distribution

**FIGURE 5:**
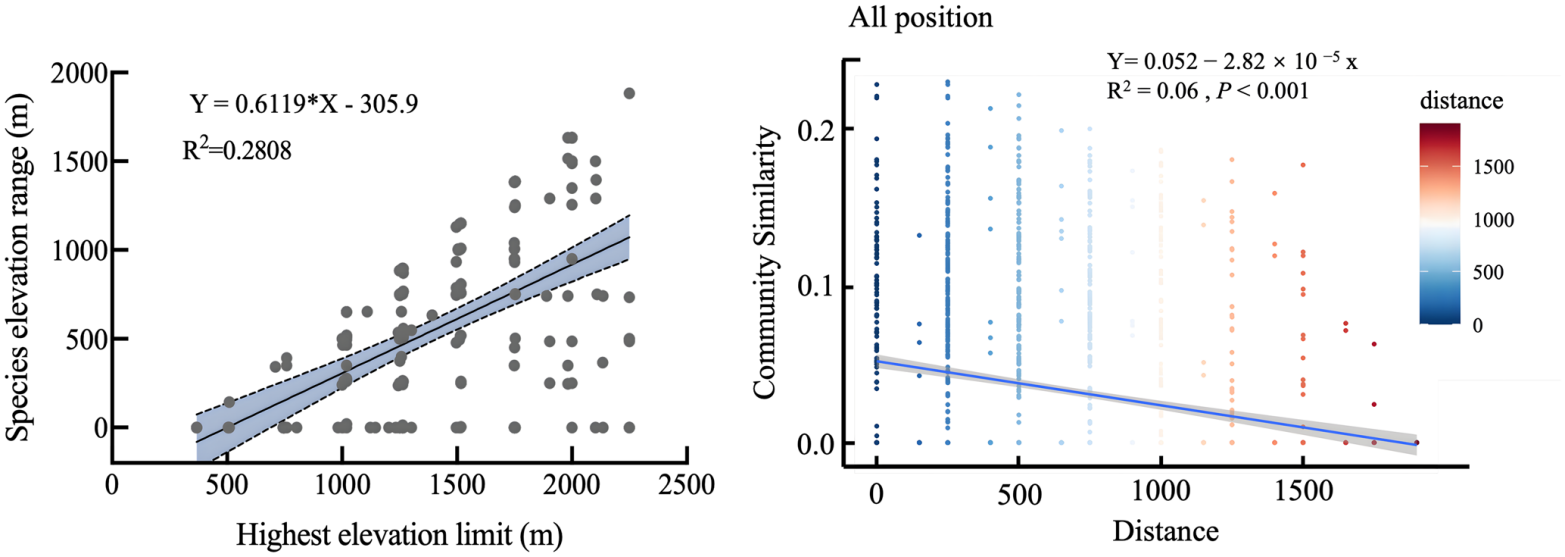
Relationship between upper elevational limits and elevational range width of recorded ant species (left), and distance‐decay relationship of ant community beta diversity across elevation (right).

Analysis of the relationship between the highest elevational limits and the species elevational range of ant species revealed a significant positive correlation (Figure 5 left), consistent with Rapoport's rule, which posits that species occurring at higher elevations exhibit broader altitudinal niche. We subsequently established DDR models for ant communities across elevational gradients. The results demonstrated that community similarity declined significantly with increasing elevational distance (*p* < 0.001) (Figure 5 right). After system analysis, we found that 103 species of ants (49.28%) have a narrow elevational distribution, 60 species of ants (28.71%) have a relatively narrow range, 38 species of ants (18.19%) have a moderate range, and only 8 species of ants (3.83%) have a relatively wide elevational adaptability range. Among them, *Prenolepis naoroji* (Forel, 1902) has the widest elevational distribution, reaching 1883 m, followed by *Ectomomyrmex javanus* (Mayr, 1867), *Brachyponera brevidorsa* (Xu, 1994), and *Pheidole tumida* (Eguchi, 2008), among which 5 ant species have an elevational distribution range of up to 1633 m. In addition, 68 species of ants were found only at one altitude (Supporting Information S2).

**FIGURE 6:**
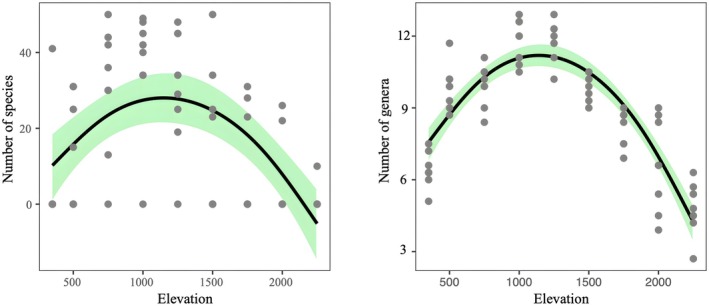
Relationships between diversity measures (number of species, number of genera) and elevation.

Mixed‐effects modeling revealed a significant unimodal distribution of ant species and genus diversity along the elevational gradient (*p* < 0.01, Figure 6), peaking at mid‐elevations around 1000 m. This pattern is likely attributed to the ecotone between tropical seasonal rainforests and coniferous‐broadleaved mixed forests at mid‐elevations, fostering greater niche heterogeneity. Notably, the Akaike Information Criterion (AIC) and Bayesian Information Criterion (BIC) values for species richness were significantly higher than those for genus‐level diversity (Table 2), indicating that the genus‐level model exhibits superior statistical robustness and ecological explanatory power in response to elevational gradients. Collectively, these findings demonstrate that mid‐elevation habitats serve as critical conservation zones for maintaining ant biodiversity in Huanglianshan National Nature Reserve.

**TABLE 2:** Statistical details of models examining relationships between elevation and diversity metrics.

Predictor variable	Response variable	*p*	*R* ^2^	AIC	BIC	Estimate1	Estimate2	F	NumDF	DenDF
Elevation	Species	< 0.001	0.313	631.7495	643.1328	0.06499	−0.0000281	23.355	1	62
Elevation	Genera	< 0.001	0.751	454.8476	466.231	0.04458	−0.0000194	131.49	1	69

### 3.3 Foraging and Nesting Sites

A total of 204 ant species with recorded foraging sites were identified (Supporting Information S3). There is a greater diversity of ant species foraging on the ground and in the soil, with 172 and 124 species, respectively, while 63 species were found foraging on plants. Among them, four ant species, including *Dolichoderus thoracicus* (Smith, 1860), *Brachyponera brevidorsa* (Xu, 1994), and *Brachyponera luteipes* (Mayr, 1862), have a wide foraging range and were found in all seven types of sites. Additionally, 70 species of ants (33.49%) were found foraging in only one type of site.

A total of 101 species of ants have been recorded with known nesting sites (Supporting Information S3). The largest number of ant species nests in soil, reaching 64 species (30.62%), followed by those nesting in rotten wood (46 species) and under stones (40 species); the fewest ants nest on plants (1 species). Among these, four ant species, including *Brachyponera brevidorsa* (Xu, 1994), *Brachyponera luteipes* (Mayr, 1862), and *Tetramorium ciliatum* (Bolton, 1977), can nest in four types of locations. Thirteen species, such as *Stictoponera bicolor* (Emery, 1889), *Odontoponera transversa* (F. Smith, 1857), and *Brachyponera xui* (Chen et al., 2025), can nest in three types of sites. The nesting sites of the remaining 108 ant species have not yet been determined.

### 3.4 Habitat

**FIGURE 7:**
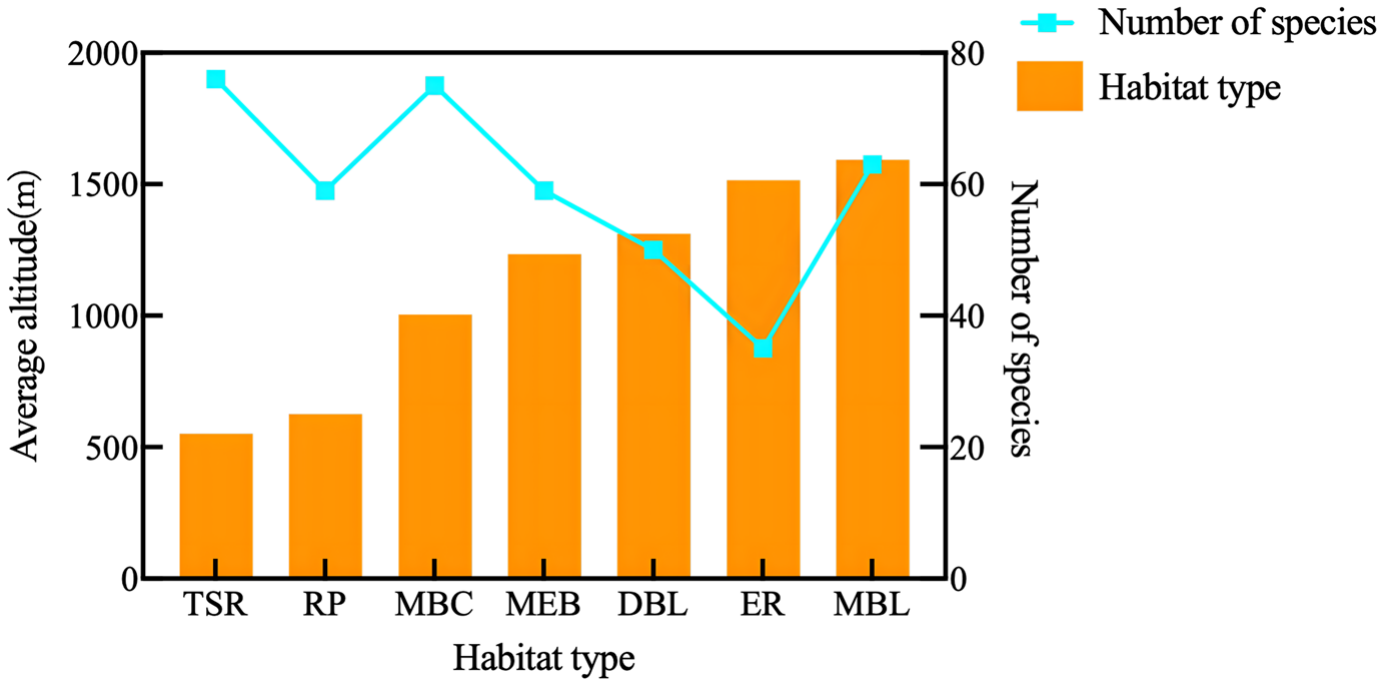
Distribution of Ant Species in Different Habitats of Huanglianshan Nature Reserve. Abbreviations denote habitat types: DBL, deciduous broad‐leaved forest; ER, Eucalyptus forest; MBC, mixed broadleaf‐conifer forest; MBL, Mountain moss evergreen broad‐leaved forest; MEB, monsoon evergreen broadleaf forest; RP, rubber plantation; TSR, tropical seasonal rainforest.

The number of ant species inhabiting the tropical seasonal rainforest and the coniferous‐broadleaf mixed forest is the richest (Figure 7), reaching 76 and 75 species, respectively; the montane mossy evergreen broadleaf forest comes next, with 63 species. The number of ant species in other habitats drops significantly; both rubber plantations and monsoon evergreen broadleaf forests have the same number of species, each with 59 species. This is followed by deciduous broadleaf forests (50 species) and eucalypt plantations (35 species). Among the 209 ant species, seven species (3.35%)—including *Brachyponera brevidorsa* (Xu, 1994), *Nylanderia bourbonica* (Forel, 1886), and *Crematogaster ferrarii* (Emery, 1887)—are found in all seven habitat types, showing the broadest habitat adaptation. Eleven species (5.26%), such as *Technomyrmex brunneus* (Forel, 1895), *Odontomachus circulus* (Wang, 1993), and *Pheidole nodus* (Smith, 1874), inhabit six habitat types; fifteen species (7.18%) live in five habitat types; and twenty‐one species (10.04%) occur in four habitat types. A total of 155 species (74.16%) are found in fewer than three habitat types, among which as many as 86 species (41.14%) are restricted to only one habitat type (Supporting Information S2).

### 3.5 Similarity of Ant Communities in Different Vertical Zones

The similarity coefficient range of ant communities in the 8 transects of Huanglian Mountain National Nature Reserve is 0.0761 to 0.5935, indicating that their levels range from extremely dissimilar to moderately similar (Table 3). This suggests that there is considerable differentiation among ant communities in different vertical zones. The average value of the Jaccard similarity coefficient is 0.3063, representing a moderate level of similarity. The similarity coefficient is highest (0.5935) between the Dagu transects at an elevation of 795 m and the Xiaoheijiang transects at 929 m. As altitude increases, there is a general trend of decreasing similarity coefficients between vertical zones. Notably, the similarity coefficients between the Laobian transects at 1749 m and the Erfu transects at 1132 m, and the Molu transects at 988 m are both below 0.1000.

**TABLE 3:** Similarity coefficients between ant communities in different transects.

Vertical zone	Average altitude (m)	Similarity coefficients *q*						
		Dagu (795)	Xiaoheijiang (929)	Moluo (988)	Dongma (1044)	Erfu (1132)	Qimaba (1267)	Hexin (1375)
Xiaoheijiang	929	0.5935						
Moluo	988	0.4915	0.4874					
Dongma	1044	0.4769	0.4692	0.5000				
Erfu	1132	0.4741	0.4370	0.4706	0.4348			
Qimaba	1267	0.3284	0.3968	0.3717	0.3934	0.2957		
Hexin	1375	0.3585	0.4211	0.3929	0.3267	0.3404	0.3957	
Laobian	1749	0.0862	0.1053	0.0938	0.1009	0.0761	0.1818	0.1508

## 4 Discussion

The Huanglianshan National Nature Reserve features a vertical relief of 2317 m, where climatic conditions and vegetation types form distinct elevational gradients, spanning a complete altitudinal zonation from tropical rainforests to alpine mossy forests (Song et al. 2014). In this study, a total of 9 subfamilies, 66 genera, and 209 species of Formicidae insects were recorded along an elevational gradient of 367–2250 m in the Huanglianshan National Nature Reserve, among which 24 undetermined species were discovered. In the low‐latitude regions of Yunnan, the species richness in Huanglianshan is slightly lower than that of the Xishuangbanna National Nature Reserve at the same latitude (10 subfamilies, 76 genera, 286 species) (Z. H. Xu 2002) and the Gaoligong Mountain National Nature Reserve (9 subfamilies, 67 genera, 245 species) (Xu et al. 2022), but higher than that of the Nangunhe Nature Reserve in Southwest China (10 subfamilies, 65 genera, 188 species) (Song et al. 2014) and the Tongbiguan Nature Reserve (10 subfamilies, 49 genera, 130 species) (Li et al. 2017). Compared to Huanglianshan's rich vertical diversity, Xishuangbanna's gentle lowland rainforests offer continuous habitats with optimal hydrothermal conditions, enhancing species dispersal. The Gaoligong Mountains form diverse climatic niches through orographic precipitation, while Nangunhe and Tongbiguan's enclosed topography with dry‐hot valleys restricts ant diversity.

Comparing eight vertical gradient transects, we found that the species number of the Laobian transects (25 species) on the northwest slope of Huanglianshan is significantly lower than that of the other seven vertical zones. This distribution pattern may be related to the unique geographical location of Huanglianshan. The Huanglianshan range generally runs in a northwest‐southeast direction. Warm and humid air currents from the Indian Ocean rise along the northwest‐southeast axis, resulting in rainfall centers on the east and west sides of the mountain, while the north side experiences insufficient precipitation due to terrain obstruction, leading to drought. This, coupled with vegetation simplification and habitat fragmentation caused by human activities, further affects the distribution. The Hexin vertical belt, which has the highest number of species (114 species, 54.54%), has the shortest survey route, forming a significant difference compared with the Laobian vertical belt, which has the longest survey route (25 species, 11.96%). The main reason is that the Hexin vertical belt covers a wider altitude range (500–2250 m), while the Laobian vertical belt is narrower (1750 m). In addition, the east–west mountain range, due to its windward slope and rainfall center, forms a water–heat‐rich and diverse habitat, whereas the northwest‐southeast direction is in the rain shadow area, resulting in habitat homogenization, leading to significant differences in ant species numbers.

Comparing eight vertical gradient transects, we found that the species richness of ants in the Huanglianshan Nature Reserve shows a unimodal curve pattern, first increasing and then decreasing with rising altitude. The number of ant species inhabiting high‐altitude vegetation types is significantly lower than that of low‐ and mid‐altitude vegetation types, indicating that altitude is an important factor affecting ant distribution. Analysis using PCoA and NMDS (stress = 0.19) demonstrates that ant community composition in the surveyed area undergoes a gradual transition with increasing elevation, where species' elevational distribution breadth shows a significant positive correlation with their highest elevational limits, conforming to Rapoport's rule and indicating a broader altitudinal niche in high‐elevation ant species. Subsequent DDR analysis reveals a significant attenuation of ant community similarity with increasing elevational distance (*p* < 0.001), corroborating both ordination approaches. These patterns fundamentally stem from synergistic interactions between elevational gradients and species adaptation strategies: primarily, elevational variation drives gradient shifts in critical ecological factors (e.g., temperature, humidity), filtering out low‐elevation specialists maladapted to extreme high‐altitude conditions, while high‐elevation species evolve broader thermal tolerance breadth through long‐term adaptation to survive across wider elevational ranges (Uhey et al. 2022). Concurrently, vegetation zonation transforms significantly with elevation (e.g., monsoon rainforest to montane mossy forest), and such habitat restructuring not only alters resource provisioning patterns but also contracts suitable habitat availability and reduces habitat heterogeneity, collectively driving the observed decline in community diversity (Schifani et al. 2024). In this study, *Prenolepis naoroji* (Forel, 1902) of the subfamily Formicinae demonstrated the strongest adaptability and was the only species found with an elevational distribution range exceeding 1800 m. Some species of this subfamily have also been identified as having a wide altitude adaptation range (1500–4250 m) in the northeastern slope of the Qinghai‐ Xizang Plateau (Chen et al., 2022), Wenshan National Nature Reserve (Cui et al. 2023), and Daweishan Nature Reserve (Yang et al. 2022). There are also significant differences in adaptability among different species within the same subfamily and genus. For example, the elevational distribution range of *Tetramorium wroughtonii* (Forel, 1902) extends from 365 to 2000 m, a difference of 1635 m, whereas *Tetramorium laparum* (Bolton, 1977) only lives at an altitude of 1250 m.

The survey found that 69 ant species (33.01%) forage in only one type of habitat, while more ant species (66.99%) choose to forage in two or more types of habitats. This may be related to ants' omnivorous nature, as foraging in multiple habitats can more effectively obtain food and expand their survival range. In the Huanglianshan Reserve, ant species foraging under trees outnumber those foraging on trees, which is consistent with other research results (Luo et al. 2019; Qian et al. 2022). The number of ant species foraging on trees is 77 (36.84%), which is higher both in absolute numbers and in proportion than in Ailao Mountain National Nature Reserve (66 species, 32.84%) (Cui et al. 2023), Wenshan National Nature Reserve (22 species, 20.21%) (Cui et al. 2023), and Daweishan National Nature Reserve (20 species, 19.23%) (Yang et al. 2022); the species number is higher but the proportion is lower than that of Eastern Daliangshan (53 species, 39.26%) (Li, Xu, Li, et al. 2023) and Central Daliangshan (46 species, 40%) (Qian et al. 2022; Chen, Xu, et al. 2022). Some studies have found that about 50% of tropical ants can forage in the tree canopy (Floren et al. 2014), while in temperate zones, only 12% of ants can reach the canopy. The proportion of ant species foraging on trees in the Huanglianshan Reserve is between these two figures, indicating that there may be a trend of increasing proportion of tree‐foraging ant species with decreasing latitude.

Ant nests are important places for ants to live and raise their offspring and are crucial to the development and expansion of ant colonies (López et al. 1992). Generally, ants mainly choose to nest in the soil, as it provides structural stability and good moisture retention (Schultheis et al. 2022). However, in the high‐altitude regions of the Qinghai‐ Xizang Plateau, ants prefer to nest under stones, mainly due to the need for heat conduction provided by stones (Chen et al. 2022). Studies have shown that the probability of ants nesting in the tree canopy is positively correlated with their heat resistance, cold resistance, and drought resistance, and negatively correlated with the colony's spatial requirements for nesting, the ability to excavate solid wood materials, foraging range, and the dominance rank within the community (Yao et al. 2021). The nesting sites of ant species in Huanglianshan are relatively single; 100 species (48.07%) nest in only one type of site, mainly in soil nests, followed by decayed wood and under‐stone nests, with only one species nesting on plants. In forest ecosystems at middle and low altitudes, soil is still the preferred nesting site for ants.

## 5 Conclusion

Huanglianshan National Nature Reserve is rich in ant species, and elevation, slope aspect, and human disturbance have significant impacts on the richness and distribution of ants. The number of ant species generally increases and then decreases with elevation, with the highest number of species found in low‐elevation tropical seasonal rainforests and coniferous‐broadleaf mixed forests, and the lowest in high‐elevation eucalypt forests. More ants forage on the ground and nest in the soil, but most ant species have small population sizes, narrow elevation tolerance, and singular foraging habitats, making them susceptible to environmental changes and human activities, which may lead to population decline and the loss of ecological functions at the regional level. Therefore, monitoring and protection should be strengthened.

## Author Contributions

**Xingze Li:** data curation (lead), formal analysis (lead), investigation (lead), software (lead), writing – original draft (lead), writing – review and editing (lead). **Huiping Zeng:** data curation (lead), formal analysis (lead), investigation (lead), software (lead). **Yanhui Zhang:** formal analysis (equal), investigation (equal), software (equal). **Yu Yu:** data curation (equal), investigation (equal), supervision (equal). **Chao Chen:** funding acquisition (lead), project administration (lead), resources (lead), supervision (lead), writing – review and editing (lead).

## Conflicts of Interest

The authors declare no conflicts of interest.

## Supporting information


**Appendix S1:** ece372404‐sup‐0001‐AppendixS1.docx.

**Appendix S2:** ece372404‐sup‐0002‐AppendixS2.xlsx.

**Appendix S3:** ece372404‐sup‐0003‐AppendixS3.xlsx.

**Appendix S4:** ece372404‐sup‐0004‐AppendixS4.docx.

## Data Availability

The data for this study is in this manuscript and in the Supporting Information S3.
