# Supplementary material for: Distribution Pattern of Ants in Huanglianshan National Nature Reserve From Yunnan, China
Source: Ecol Evol. 2025 Nov 4;15(11):e72404. doi: 10.1002/ece3.72404 (PMC12585183; doi:10.1002/ece3.72404)
Supplement: Supplementary file 1 — Appendix S1: ece372404‐sup‐0001‐AppendixS1.docx. [file ECE3-15-e72404-s003.docx]

| **Species** |
| --- |
| 1. **Dolichoderinae Forel, 1878** |
| 1. Chronoxenus wroughtonii (Forel, 1895) |
| 1. Dolichoderus affinis Emery, 1889 |
| 1. Dolichoderus hls01 |
| 1. Dolichoderus squamanodus Xu, 2001 |
| 1. Dolichoderus taprobanae (Smith, 1858) |
| 1. Dolichoderus thoracicus (Smith, 1860) |
| 1. Iridomyrmex anceps (Roger, 1863) |
| 1. Ochetellus glaber (Mayr, 1862) |
| 1. Tapinoma indicum Forel, 1895 |
| (10) Tapinoma melanocephalum (Fabricius, 1793) |
| (11) Technomyrmex bicolor Emery, 1893 |
| (12) Technomyrmex brunneus Forel, 1895 |
| (13) Technomyrmex elatior Forel, 1902 |
| (14) Technomyrmex obscurior Wheeler, 1928 |
| (15) Technomyrmex pratensis (Smith, 1860) |
| 1. **Dorylinae Bolton, 1990** |
| (16) Aenictus artipus Wilson, 1964 |
| (17) Aenictus binghamii Forel, 1900 |
| (18) Aenictus dentatus Forel, 1911 |
| (19) Aenictus hodgsoni Forel, 1901 |
| (20) Aenictus maneerati Jaitrong & Yamane, 2013 |
| (21) Aenictus parahuonicus Jaitrong & Yamane, 2011 |
| (22) Aenictus watanasiti Jaitrong & Yamane,2013 |
| (23) Aenictus yangi Liu et al., 2015 |
| (24) Cerapachys sulcinodis Emery, 1889 |
| (25) Dorylus orientalis Westwood, 1835 |
| (26) Lioponera longitarsus Mayr, 1879 |
| (27) Lioponera hls01 |
| (28) Syscia chinensis Chen, 2025 |
| 1. **Ectatomminae Emery, 1895** |
| (29) Stictoponera bicolor (Emery, 1889) |
| (30) *Stictoponera binghamii* (Forel, 1900) |
| (31) Stictoponera coccina (Zhou, 2001) |
| 1. **Formicinae Lepeletier, 1836** |
| (32) Anoplolepis gracilipes (Smith, 1857) |
| (33) Camponotus anningensis Wu and Wang,1989 |
| (34) Camponotus itoi Forel, 1912 |
| (35) Camponotus jianghuaensis Xiao & Wang, 1989 |
| (36) Camponotus kiusiuensis Santschi, 1937 |
| (37) Camponotus lasiselene Wang & Wu, 1994 |
| (38) Camponotus leucodiscus Wheeler, 1919 |
| (39) Camponotus mitis (Smith, 1858) |
| (40) Camponotus mutilarius Emery, 1893 |
| (41) Camponotus nicobarensis Mayr, 1865 |
| (42) Camponotus parius Emery,1889 |
| (43) Camponotus selene (Emery, 1889) |
| (44) Camponotus singularis (Smith, 1858) |
| (45) Camponotus hls01 |
| (46) Camponotus hls02 |
| (47) Camponotus hls03 |
| (48) Camponotus tonkinus Santschi, 1925 |
| (49) Colobopsis aurata (Karavaiev, 1935) |
| (50) Colobopsis *longi* (Forel, 1902) |
| (51) Colobopsis hls02 |
| (52) Echinopla cherapunjiensis Bharti & Gul, 2012 |
| (53) Formica cunicularia Latreille, 1798 |
| (54) Lasius coloratus Santschi, 1937 |
| (55) Lepisiota pulchella (Forel, 1892) |
| (56) Lepisiota reticulata Xu, 1994 |
| (57) Lepisiota rothneyi (Forel, 1894) |
| (58) Myrmoteras binghamii Forel,1893 |
| (59) Nylanderia amia (Forel, 1913) |
| (60) Nylanderia birmana (Forel, 1902) |
| (61) Nylanderia bourbonica (Forel, 1886) |
| (62) Nylanderia emmae (Forel, 1894) |
| (63) Nylanderia flavipes (Smith, 1874) |
| (64) Nylanderia opisopthalmia (Zhou & Zheng, 1998) |
| (65) Nylanderia taylori (Forel, 1894) |
| (66) Oecophylla smaragdina (Fabricius, 1775) |
| (67) Paraparatrechina hls01 |
| (68) Paraparatrechina aseta (Forel, 1902) |
| (69) Paraparatrechina neela Sahanashree et al., 2024 |
| (70) Paraparatrechina sauteri (Forel, 1913) |
| (71) Paratrechina longicornis (Latreille, 1802) |
| (72) Polyrhachis armata (Le Guilliou, 1841) |
| (73) Polyrhachis bicolor Smith, 1858 |
| (74) Polyrhachis burmanensis Donisthorpe, 1938 |
| (75) Polyrhachis halidayi Emery, 1889 |
| (76) Polyrhachis hippomanes Smith, 1861 |
| (77) Polyrhachis illaudata Walker, 1859 |
| (78) Polyrhachis latona Wheeler, 1909 |
| (79) Polyrhachis rastellata (Latreille, 1802) |
| (80) Polyrhachis rufipes Smith, 1858 |
| (81) Polyrhachis striata Mayr, 1862 |
| (82) Polyrhachis tibialis Smith, 1858 |
| (83) Prenolepis fustinoda Williams & LaPolla, 2016 |
| (84) Prenolepis melanogaster Emery, 1893 |
| (85) Prenolepis naoroji Forel, 1902 |
| (86) Prenolepis shanialena Williams and LaPolla, 2016 |
| (87) Prenolepis striata Chen & Zhou, 2018 |
| (88) Pseudolasius emeryi Forel,1911 |
| (89) Pseudolasius silvestrii Wheeler, 1927 |
| (90) Pseudolasius hls01 |
| (91) Pseudolasius zamrood Akbar et al., 2017 |
| 1. **Leptanillinae Emery, 1910** |
| (92) Protanilla bicolor Xu, 2002 |
| 1. **Myrmicinae Lepeletier, 1836** |
| (93) Aphaenogaster beccarii Emery,1887 |
| (94) Aphaenogaster feae Emery, 1889 |
| (95) Aphaenogaster lepida Wheeler, 1930 |
| (96) Aphaenogaster pumilopuncta Zhou, 2001 |
| (97) Aphaenogaster hls01 |
| (98) Cardiocondyla itsukii Seifert et al., 2017 |
| (99) Cardiocondyla kagutsuchi Terayama, 1999 |
| (100) Cardiocondyla nuda (Mayr, 1866) |
| (101) Cardiocondyla obscurior Wheeler, 1929 |
| (102) Cardiocondyla hls01 |
| (103) Cardiocondyla wroughtonii (Forel, 1890) |
| (104) Carebara affinis (Jerdon, 1851) |
| (105) Carebara altinoda (Xu,2003) |
| (106) Carebara obtusidenta (Xu, 2003) |
| (107) Carebara hls01 |
| (108) Carebara trechideros (Zhou & Zheng, 1997) |
| (109) Cataulacus granulatus (Latreille, 1802) |
| (110) Crematogaster anthracina Smith, 1857 |
| (111) Crematogaster ferrarii Emery, 1888 |
| (112) Crematogaster macaoensis Wu & Wang, 1995 |
| (113) Crematogaster rogenhoferi Mayr, 1878 |
| (114) Crematogaster rothneyi Mayr, 1879 |
| (115) Crematogaster treubi Emery, 1896 |
| (116) Crematogaster zoceensis Santschi, 1925 |
| (117) *Dilobocondyla* hls01 |
| (118) Kartidris ashima Xu et Zheng,1995 |
| (119) Kartidris matertera Bolton, 1991 |
| (120) Lophomyrmex birmanus Emery, 1893 |
| (121) Meranoplus laeviventris Emery, 1889 |
| (122) Monomorium chinense Santschi, 1925 |
| (123) Monomorium orientale Mayr,1879 |
| (124) Monomorium pharaonis (Linnaeus, 1758) |
| (125) Monomorium triviale Wheeler, 1906 |
| (126) Myrmecina guangxiensis Zhou, 2001 |
| (127) Myrmecina taiwana Terayama, 1985 |
| (128) Myrmica margaritae Emery, 1889 |
| (129) Myrmica hls01 |
| (130) Myrmicaria hls01 |
| (131) Pheidole capellinii Emery, 1887 |
| (132) Pheidole constanciae Forel, 1902 |
| (133) Pheidole fortis Eguchi, 2006 |
| (134) Pheidole hongkongensis Wheeler, 1928 |
| (135) Pheidole nodus Smith, 1874 |
| (136) Pheidole pieli Santschi, 1925 |
| (137) Pheidole plagiaria Smith, 1860 |
| (138) Pheidole planifrons Santschi, 1920 |
| (139) Pheidole roberti Forel, 1902 |
| (140) Pheidole sagei Forel, 1902 |
| (141) Pheidole smythiesii Forel, 1902 |
| (142) Pheidole hls01 |
| (143) Pheidole tumida Eguchi, 2008 |
| (144) Pheidole vulgaris Eguchi, 2006 |
| (145) Pristomyrmex brevispinosus Emery, 1887 |
| (146) Pristomyrmex punctatus (Smith, 1860) |
| (147) Recurvidris recurvispinosa (Forel, 1890) |
| (148) Solenopsis jacoti Wheeler, 1923 |
| (149) Strumigenys lewisi Cameron, 1886 |
| (150) Temnothorax angulohumerus Zhou et al., 2010 |
| (151) Tetramorium aptum Bolton,1977 |
| (152) Tetramorium bicarinatum (Nylander, 1846) |
| (153) Tetramorium ciliatum Bolton, 1977 |
| (154) Tetramorium indosinense Wheeler, 1927 |
| (155) Tetramorium kraepelini Forel, 1905 |
| (156) Tetramorium laparum Bolton, 1977 |
| (157) Tetramorium nipponense Wheeler, 1928 |
| (158) Tetramorium obtusidens Viehmeyer, 1916 |
| (159) Tetramorium parvispinum (Emery, 1893) |
| (160) Tetramorium hls01 |
| (161) Tetramorium hls03 |
| (162) Tetramorium hls04 |
| (163) Tetramorium hls05 |
| (164) Tetramorium tonganum Mayr, 1870 |
| (165) Tetramorium wroughtonii (Forel, 1902) |
| (166) Vollenhovia emeryi Wheeler, 1906 |
| (167) Vollenhovia hls01 |
| 1. **Ponerinae Lepeletier, 1836** |
| (168) Brachyponera brevidorsa Xu, 1994 |
| (169) Brachyponera candida Chen et al., 2025 |
| (170) Brachyponera luteipes (Mayr, 1862) |
| (171) Brachyponera myops Chen et al., 2025 |
| (172) Brachyponera paraarcuata Chen et al., 2025 |
| (173) Brachyponera xui Chen et al., 2025 |
| (174) Buniapone amblyops (Emery,1887) |
| (175) *Centromyrmex feae* (Emery,1889) |
| (176) *Diacamma rugosum* (Le Guillou, 1842) |
| (177) *Ectomomyrmex annamitus* (André, 1892) |
| (178) Ectomomyrmex javanus Mayr, 1867 |
| (179) *Ectomomyrmex leeuwenhoeki* (Forel, 1886) |
| (180) Ectomomyrmex zhengi (Xu, 1995) |
| (181) *Euponera pilosior* Wheeler, 1928 |
| (182) Euponera hls01 |
| (183) *Harpegnathos venator* (Smith, 1858) |
| (184) *Hypoponera confinis* (Roger, 1860) |
| (185) *Hypoponera sauteri* Wheeler, 1929 |
| (186) Hypoponera hls01 |
| (187) Hypoponera hls02 |
| (188) *Leptogenys birmana* Forel, 1900 |
| (189) Leptogenys kitteli (Mayr, 1870) |
| (190) *Leptogenys lucidula* Emery, 1895 |
| (191) Leptogenys hls01 |
| (192) *Mesoponera* hls01 |
| (193) Myopias hania Xu & Liu, 2012 |
| (194) *Myopias sonthichaiae* Jaitrong et al., 2018 |
| (195) *Odontomachus circulus* Wang, 1993 |
| (196) *Odontomachus granatus* Wang, 1993 |
| (197) *Odontomachus rixosus* Smith, 1857 |
| (198) *Odontoponera denticulata* (Smith, 1858) |
| (199) *Odontoponera transversa* (Smith, 1857) |
| (200) *Ponera guangxiensis* Zhou, 2001 |
| (201) *Ponera sinensis* Wheeler, 1928 |
| (202) Ponera hls02 |
| (203) *Pseudoneoponera rufipes* (Jerdon, 1851) |
| 1. **Proceratiinae Emery, 1895** |
| (204) *Proceratium deelemani* Perrault, 1981 |
| 1. **Pseudomyrmecinae Smith, 1952** |
| (205) *Tetraponera allaborans* (Walker, 1859) |
| (206) *Tetraponera attenuata* Smith, 1877 |
| (207) *Tetraponera convexa* Xu & Chai, 2004 |
| (208) *Tetraponera nitida* (Smith, 1860) |
| (209) *Tetraponera rufonigra* (Jerdon, 1851) |
