# Supplementary material for: Distribution Pattern of Ants in Huanglianshan National Nature Reserve From Yunnan, China
Source: Ecol Evol. 2025 Nov 4;15(11):e72404. doi: 10.1002/ece3.72404 (PMC12585183; doi:10.1002/ece3.72404)
Supplement: Supplementary file 4 — Appendix S4: ece372404‐sup‐0004‐AppendixS4.docx. [file ECE3-15-e72404-s001.docx]

Supplementary Material 4:Â Scripts for Plotting Figures

**PCoA（Figure 4 left）**

############ PCoA分析 16S扩增子数据 ######

rm(list=ls()) #清空当前工作环境

###当前工作目录

getwd()

###加载包

library(microeco)

library(magrittr)

library(ggplot2)

library(openxlsx)

theme_set(theme_bw())

####1. 导入数据####

otu_raw <- read.xlsx("otu.xlsx", rowNames = TRUE)

groups <- read.xlsx("group.xlsx", rowNames = TRUE)

groups$Sample <- rownames(groups) #增加列名至最后1列

tax <- read.xlsx("tax.xlsx", rowNames = TRUE)

tax %<>% tidy_taxonomy

####2.创建dataset数据集#####

dataset <- microtable$new(sample_table = groups, otu_table = otu_raw, tax_table = tax, auto_tidy = T)

####3. PCoA分析####

dataset$cal_betadiv()

t1 <- trans_beta$new(dataset = dataset, group = "Group", measure = "bray")

t1$cal_ordination(method = "PCoA")

# 保存PCoA结果

write.table(t1$res_ordination$scores, file = "beta/Group/PCoA_res_ordination_scores.txt",

sep = "\t", quote = F, row.names = T)

write.table(t1$res_ordination$eig, file = "beta/Group/PCoA_res_ordination_eig.txt",

sep = "\t", quote = F, row.names = T)

####4. 绘制PCoA图####

p <- t1$plot_ordination(plot_color = "Group", plot_type = c("point", "chull"))

p0 <- t1$plot_ordination(plot_color = "Group", plot_type = c("point", "ellipse"))

# 保存图形

ggsave(p, file = 'beta/Group/PCoA_bray.pdf', width = 4, height = 3)

ggsave(p0, file = 'beta/Group/PCoA_bray1.pdf', width = 4, height = 3)

ggsave(p, file = 'beta/Group/PCoA_bray.tiff', width = 4, height = 3)

ggsave(p0, file = 'beta/Group/PCoA_bray1.tiff', width = 4, height = 3)

print(p)

print(p0)

NMDS（Figure 4 right）

############ NMDS分析 16S扩增子数据 ######

rm(list=ls()) #清空当前工作环境

###当前工作目录

getwd()

###加载包

library(microeco)

library(magrittr)

library(ggplot2)

library(openxlsx)

theme_set(theme_bw())

####1. 导入数据####

otu_raw <- read.xlsx("otu.xlsx", rowNames = TRUE)

groups <- read.xlsx("group.xlsx", rowNames = TRUE)

groups$Sample <- rownames(groups) #增加列名至最后1列

tax <- read.xlsx("tax.xlsx", rowNames = TRUE)

tax %<>% tidy_taxonomy

####2.创建dataset数据集#####

dataset <- microtable$new(sample_table = groups, otu_table = otu_raw, tax_table = tax, auto_tidy = T)

####3. NMDS分析####

dataset$cal_betadiv()

t1 <- trans_beta$new(dataset = dataset, group = "Group", measure = "bray")

t1$cal_ordination(method = "NMDS")

# 重命名坐标轴

names(t1$res_ordination$scores)[1:2] <- c('NMDS1', 'NMDS2')

####4. 绘制NMDS图####

p <- t1$plot_ordination(plot_color = "Group", plot_type = c("point")) + theme_bw()

# 保存图形

ggsave(p, file = 'beta/NMDS_bray.pdf', width = 4, height = 3)

ggsave(p, file = 'beta/NMDS_bray.tiff', width = 4, height = 3)

####5. 统计检验####

# perMANOVA分析

t1$cal_manova(manova_all = TRUE)

write.table(t1$res_manova, file = "beta/Group/对所有样本进行manova分析.txt",

sep = "\t", quote = F, row.names = T)

t1$cal_manova(manova_all = FALSE)

write.table(t1$res_manova, file = "beta/Group/对每对样品分组间进行manova分析.txt",

sep = "\t", quote = F, row.names = T)

# Betadisper检验

t1$cal_betadisper()

output <- capture.output(print(t1$res_betadisper))

writeLines(output, "beta/Group/对每对样品分组间进行manova分析_res_betadisper.txt")

# ANOSIM检验

t1$cal_anosim(group = "Group")

output <- capture.output(print(t1$res_anosim))

writeLines(output, "beta/Group/t1$res_anosim_total.txt")

t1$cal_anosim(group = "Group", paired = TRUE)

output <- capture.output(print(t1$res_anosim))

writeLines(output, "beta/Group/t1$res_anosim.txt")

# 保存数据

save(t1, file = "beta/Group/t1_NMDS.RData")

print(p)

Distance-decay relationship (Figure 5 right)

rm(list=ls())

library(rstudioapi)

# Get the name of the directory in which the current file is located.

cur_dir = dirname(getSourceEditorContext()$path)

# 加载R包

library(ggplot2)

library(geosphere)

library(fdrtool)

library(vegan)

library(maps)

library(ggpubr)

library(ggpmisc)

# Change the working directory to the directory with the current file.

setwd(cur_dir)

# 读取文件

otu <- read.csv("otu_sediment.csv",header = T,row.names = 1)

env <- read.csv("sediment.csv",header = T,row.names = 1)

common_samples <- intersect(colnames(otu), rownames(env))

otu <- otu[, common_samples]

env <- env[common_samples, ]

euclidean_dist <- vegdist(env, method = "euclidean")

plot_map<-data.frame(as.vector(1-vegdist(t(otu))),as.vector(as.dist(euclidean_dist)))

names(plot_map)<-c("similarity","distance")

#进行拟合分析观察距离和微生物群落相似性的变化趋势

p1<- ggplot(plot_map, aes(x=distance, y=similarity, color=distance))+

geom_jitter(position=position_jitter(0.17), size=1.5, alpha=1)+theme_classic() +

labs(x="Distance", y="Community Similarity")+theme_bw()+

geom_smooth(method=lm,level=0.95,size=1.1,se=T,color='#006FB0')+#拟合线

scale_color_gradientn(colours = rev(RColorBrewer::brewer.pal(11,"RdBu")))+

theme_bw(base_line_size = 1.05,base_rect_size = 1.05)+

theme(panel.grid.major=element_blank(),panel.grid.minor=element_blank())+

theme(axis.text=element_text(colour='black',size=9))+

geom_smooth(method = 'lm', formula = y~x, se = TRUE, show.legend = FALSE, alpha=0.10) +

stat_poly_eq(aes(label = paste(..eq.label.., ..rr.label.., stat(p.value.label),sep = '~`,`~')),

formula = y~x, parse = TRUE, label.x = 0.90, label.y = 0.90, size = 3)+

ggtitle("All position")

p1

ggsave("./distance_decay_output/dist_decay.png",dpi = 600,width = 7,height = 4)

#############################

# 首先拟合线性模型

model <- lm(similarity ~ distance, data = plot_map)

# 提取R平方值

r_squared <- summary(model)$r.squared

# 打印R平方值

print(paste("R-squared:", round(r_squared, 4)))

# 如果您想要更完整的模型摘要

summary(model)

mixed-effects model（Figure 6）

library(lme4) # 拟合混合效应模型

library(ggplot2) # 绘图

library(ggeffects) # 生成预测值（推荐）

library(splines)

library(lmerTest) # 用于获取p值

library(performance) # 用于R2计算

# 如果未安装ggeffects，先运行: install.packages("ggeffects")

data <- read.csv("data.csv",header = T,row.names = 1)

model <- lmer(

species ~ Hight + I(Hight^2) + (1 | block),

data = data

)

summary(model)

anova(model)

r2_nakagawa <- r2(model)

print(r2_nakagawa)

AIC(model)

BIC(model)

pred <- ggpredict(model, terms = c("Hight [all]")) # 对所有Hight值预测

head(pred) # 查看预测数据框

# 3. 绘制图形（自定义样式）

ggplot() +

# 浅绿色置信区间（透明度0.6，无边框）

geom_ribbon(

data = pred,

aes(x = x, ymin = conf.low, ymax = conf.high),

fill = "#90EE90", # 浅绿色

alpha = 0.6, # 透明度

color = NA # 无边框线

) +

# 黑色回归曲线

geom_line(

data = pred,

aes(x = x, y = predicted),

color = "black",

linewidth = 1

) +

# 灰色数据点（不显示图例）

geom_point(

data = data,

aes(x = Hight, y = species),

color = "gray50", # 灰色

size = 2,

alpha = 0.6,

show.legend = FALSE # 隐藏图例

) +

# 坐标轴和标题

labs(

title = "物种数量与海拔的关系",

x = "海拔 (m)",

y = "物种数量"

) +

# 简洁主题

theme_bw() +

theme(legend.position = "none") # 确保无图例

###############################平滑

pred <- ggpredict(

model,

terms = c("Hight [n=1000]") # 增加预测点密度

)

# （可选）用样条插值进一步平滑曲线

hight_smooth <- seq(min(data$Hight), max(data$Hight), length.out = 1000)

smooth_curve <- data.frame(

x = hight_smooth,

y = predict(

lm(predicted ~ ns(x, df = 5), data = pred), # 自然样条插值

newdata = data.frame(x = hight_smooth)

),

conf.low = approx(pred$x, pred$conf.low, xout = hight_smooth)$y, # 插值置信区间

conf.high = approx(pred$x, pred$conf.high, xout = hight_smooth)$y

)

# ---------------------------

# 3. 绘制图形（完全自定义样式）

# ---------------------------

ggplot() +

# 浅绿色置信区间（半透明，无边框）

geom_ribbon(

data = smooth_curve, # 使用插值后的数据

aes(x = x, ymin = conf.low, ymax = conf.high),

fill = "#90EE90", # 浅绿色

alpha = 0.6, # 透明度

color = NA # 无边框线

) +

# 黑色回归线（平滑后）

geom_line(

data = smooth_curve,

aes(x = x, y = y),

color = "black",

linewidth = 1.2 # 稍粗的线条

) +

# 灰色原始数据点（无图例）

geom_point(

data = data,

aes(x = Hight, y = species),

color = "gray50", # 中性灰

size = 2,

alpha = 1, # 适度透明

show.legend = FALSE # 隐藏图例

) +

# 坐标轴和标题

labs(

title = "物种数量与海拔的关系",

x = "海拔 (m)",

y = "物种数量"

) +

# 简洁主题（无图例、无背景网格）

theme_bw() +

theme(

legend.position = "none", # 彻底移除图例

panel.grid.major = element_blank(), # 可选：移除主网格线

panel.grid.minor = element_blank() # 可选：移除次网格线

)

#####################参数
